# Fungal Communities in Sediments Along a Depth Gradient in the Eastern Tropical Pacific

**DOI:** 10.3389/fmicb.2020.575207

**Published:** 2020-11-06

**Authors:** Keilor Rojas-Jimenez, Hans-Peter Grossart, Erik Cordes, Jorge Cortés

**Affiliations:** ^1^Escuela de Biología, Universidad de Costa Rica, San José, Costa Rica; ^2^Institute for Biochemistry and Biology, University of Potsdam, Potsdam, Germany; ^3^Department of Experimental Limnology, Leibniz-Institute of Freshwater Ecology and Inland Fisheries, Stechlin, Germany; ^4^Department of Biology, Temple University, Philadelphia, PA, United States; ^5^Centro de Investigación en Ciencias del Mar y Limnología, Universidad de Costa Rica, San José, Costa Rica

**Keywords:** deep-sea, aquatic fungi, biodiversity, *Metschnikowia*, Costa Rica

## Abstract

Deep waters represent the largest biome on Earth and the largest ecosystem of Costa Rica. Fungi play a fundamental role in global biogeochemical cycling in marine sediments, yet, they remain little explored. We studied fungal diversity and community composition in several marine sediments from 16 locations sampled along a bathymetric gradient (from a depth of 380 to 3,474 m) in two transects of about 1,500 km length in the Eastern Tropical Pacific (ETP) of Costa Rica. Sequence analysis of the V7-V8 region of the 18S rRNA gene obtained from sediment cores revealed the presence of 787 fungal amplicon sequence variants (ASVs). On average, we detected a richness of 75 fungal ASVs per sample. Ascomycota represented the most abundant phylum with Saccharomycetes constituting the dominant class. Three ASVs accounted for ca. 63% of all fungal sequences: the yeast *Metschnikowia* (49.4%), *Rhizophydium* (6.9%), and *Cladosporium* (6.7%). We distinguished a cluster composed mainly by yeasts, and a second cluster by filamentous fungi, but we were unable to detect a strong effect of depth and the overlying water temperature, salinity, dissolved oxygen (DO), and pH on the composition of fungal communities. We highlight the need to understand further the ecological role of fungi in deep-sea ecosystems.

## Introduction

Fungi inhabited the oceans, including the deep-sea ecosystem, long before they conquered terrestrial environments. In addition, considering that the deep sea represents the largest biome on Earth, there is a paucity of studies on the diversity and ecology of fungi in this ecosystem compared to the rest of the ocean. Furthermore, what is known about the microbial ecology in deep-sea sediments is mainly about bacteria and archaea (Edgcomb et al., 2011; Nagano and Nagahama, 2012; Dekas et al., 2016; Xu et al., 2018). Therefore, detailed knowledge of deep-sea fungi is required to understand better the overall fungal contribution to marine food webs and biogeochemical cycles at the global scale (Manohar and Raghukumar, 2013; Barone et al., 2018; Drake and Ivarsson, 2018; Grossart et al., 2019; Román et al., 2019; Hassett et al., 2020).

Fungal communities have been studied in only a small part of the great variety of habitats that exist in deep waters. Some of these habitats include sediments of hydrothermal vents, methane-cold seeps, oxygen-minimum zones, and associated with other macro-organisms (Nagahama et al., 2011; Zhang et al., 2016; Batista-García et al., 2017). In addition, some studies have shown that the subseafloor represents a vast ecosystem where micro-aerobic respiration occurs and where microbial life subsist, even hundreds of meters below the seafloor (D’Hondt, 2002; Roy et al., 2012; D’Hondt et al., 2015; Ivarsson et al., 2016a; Nagano et al., 2016).

In recent years, there has been a growing interest in studying fungal communities in deep-sea environments using culture-dependent and, to an increasing extent, culture-independent methods. Abundant fungal populations have been observed in a variety of deep-sea locations such as asphalt seeps in São Paulo Plateau (Nagano et al., 2017), methane seeps in the Kuroshima Knoll (Takishita et al., 2006), hydrothermal vents in the Mid-Atlantic Ridge (Le Calvez et al., 2009; Xu et al., 2017), sediments of the Peru Trench (Edgcomb et al., 2011), the East Indian Ocean (Zhang et al., 2014), the High Arctic (Zhang et al., 2015), the Mariana Trench (Xu et al., 2016, 2018), the Yellow Sea (Li et al., 2016), the Mediterranean Sea (Barone et al., 2018), the Yap Trench (Li et al., 2019), and subsurface sediments in Suruga-Bay (Nagano et al., 2016).

In general, Ascomycota and Basidiomycota are the most abundant groups in deep-sea ecosystems, representing between 70–80% and 10–20% of the sequences, respectively. Some of the most abundant filamentous fungal genera include *Penicillium*, *Aspergillus*, *Cladosporium*, and *Fusarium*, while some of the most abundant yeasts include *Rhodotorula*, *Cryptococcus*, *Candida*, *Rhodosporidium*, and *Metschnikowia* (Li et al., 2016, 2019; Xu et al., 2016, 2019; Zhang et al., 2016; Nagano et al., 2017; Barone et al., 2018; Wang et al., 2019).

In deep waters, fungi must be adapted to the total absence of light, low temperatures, and high hydrostatic pressure. Fungi in the deep-sea sediments may survive on marine snow, which consists of organic matter derived from photosynthesis that takes place in the photic layer (Bochdansky et al., 2017). In addition to performing aerobic respiration, fungi could be capable of carrying out processes such as fermentation, sulfate reduction, methanogenesis (D’Hondt, 2002; Lenhart et al., 2012), and possibly lithoautotrophy (López-García et al., 2003; Nealson et al., 2005; Ivarsson et al., 2016b). Transcriptomic analyses also confirm fungi as active members of deep-sea sediments, performing activities related to complex carbon and fatty acid metabolism (Pachiadaki et al., 2016). These metabolic processes may be more critical for fungi in deep waters since it has been observed that as depth increases, fungal populations exhibit a more multitrophic lifestyle (Li et al., 2019).

Considering the enormous area to be explored for fungal diversity and function in deep-sea sediments, the existing studies are minimal and often lack an adequate spatial and temporal resolution (Grossart and Rojas-Jimenez, 2016; Grossart et al., 2019; Morales et al., 2019). Therefore, there is still a large number of geographical locations that have not yet been studied, including the Eastern Tropical Pacific (ETP). The deep-sea waters of the ETP constitute a particularly important ecosystem in Costa Rica since they represent about 90% of the whole territory (Cortés, 2016, 2019).

The Costa Rican ETP comprises a chain of mountains and submarine volcanoes across the subduction zone of the Cocos and Caribbean tectonic plates. Here, there is a high diversity of microhabitats (Lizano, 2001; Protti et al., 2012; Rojas and Alvarado, 2012) including methane seeps (Sahling et al., 2008; Levin et al., 2012, 2015). Previous studies have shown high endemism and diversity of macro- and microorganisms in this region (Rusch et al., 2007; Cortés, 2008, 2019; Rojas-Jiménez, 2018). Also, the Costa Rican ETP is part of a marine corridor that extends through Isla del Coco to the Galapagos Islands in Ecuador, which represents an essential site for the conservation and regeneration of marine species throughout the ETP (Cortés, 2012).

In this work, we have explored the diversity and composition of fungal communities in deep-sea sediments of the Costa Rican ETP. Two expeditions were carried out with transects of approximately 1,500 km length each, and sediments were sampled at 16 locations at depths between 380 m and 3,474 m. We extracted DNA from subsamples of each sediment core, sequenced the 18S rRNA gene, and performed a subsequent bioinformatic analysis. This work confirms the high abundance and diversity of fungi in sediments of the ETP region. We expect that our results will support current efforts to conserve this region by providing a baseline of the high diversity of fungal species and microhabitats found in its deep-sea waters.

## Materials and Methods

**FIGURE 1:**
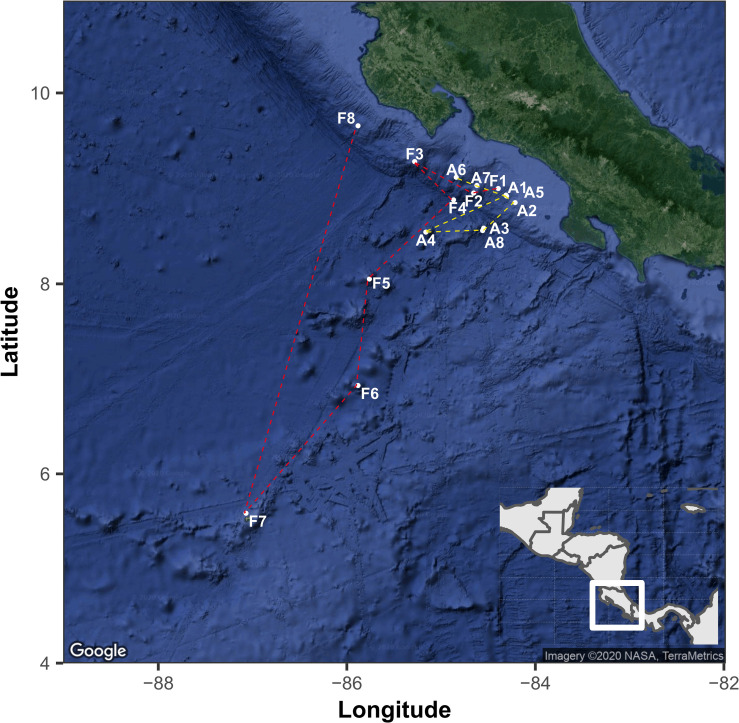
The geographical location of sampling points in the Eastern Tropical Pacific of Costa Rica. The points indicated with the letter A and yellow line correspond to the route followed by the RV Atlantis and while letter F and red lines correspond to the route of RV Falkor. The map was generated with the ggmap package using a Google satellite image.

We analyzed the fungal community composition in sediments along a depth gradient in the ETP of Costa Rica, across two transects of ca. 1,500 km length each (Figure 1). All samples were collected with the permission of the Ministry of Environment and Energy of Costa Rica (SINAC-CUSBSE-PI-R-032-2018; R-070-2018-OT-CONAGEBIO). The RV *Atlantis* surveyed the Pacific continental margin of Costa Rica from October 24th to November 5th, 2018, from the continental slope to the offshore seamounts across a subduction zone. In this region, several methane-rich seeps have been detected (Sahling et al., 2008; Levin et al., 2012, 2015). All sediment cores were collected by the human-occupied vehicle (HOV) *Alvin* equipped with mechanical, maneuverable arms. We analyzed eight sediment-cores from this expedition. The following year, the RV *Falkor* surveyed the seamounts extending from the mainland to the Isla del Coco National Park between January 6th and 21st, 2019. This region comprises several seamounts and natural gas seeps and provides an important corridor for highly specialized biological communities occupying the area. The sediment cores were collected employing the remotely operated vehicle (ROV) *SuBastian*, which is also equipped with mechanical, maneuverable arms. We analyzed another eight sediment cores from this expedition. The cores consist of an acrylic sleeve (6.7 cm diameter by 25.4 cm long) with a PVC cap and a rubber flap on the top to allow for water to escape while inserting the core while sealing as the core is removed from the sediment. The cores were kept in a “quiver” which is a PVC sleeve with a stopper at the bottom. They are sealed to the outside and are not contaminated by seawater on the way to the surface. Because the cores traveled from a higher pressure to a lower pressure, we rule out seawater intrusion. The transit time of the ROV on the longest recovery (>3,200 m depth) was approximately 2 h. Further details of the sampling sites, dates, depth, temperature, salinity, dissolved oxygen (DO), and pH are shown in Table 1.

**TABLE 1:** Sites of the Eastern Tropical Pacific of Costa Rica sampled in this study, with the respective values of the environmental variables measured.

**Sample**	**RV**	**Site**	**Date**	**Depth (m)**	**Temperature (°C)**	**Salinity (PSU)**	**DO (mg/L)**	**pH**	Data sources*
A1	Atlantis	Mound 12**	24/10/18	996	5.06	34.57	1.10	7.62	1, 6
A2	Atlantis	Quepos slide**	25/10/18	380	11.75	34.76	0.20	7.71	1, 7
A3	Atlantis	Quepos plateau	26/10/18	2,200	2.06	34.60	3.73	8.06	2, 4, 6
A4	Atlantis	Seamount 3	28/10/18	1,383	3.35	34.60	1.67	7.70	2, 4, 6
A5	Atlantis	Mound 11**	3/11/18	1,024	4.83	34.57	1.24	7.67	6, 7
A6	Atlantis	Jaco scar**	4/11/18	1,788	2.54	34.63	2.42	7.61	1, 6
A7	Atlantis	Parrita seep**	5/11/18	1,410	3.41	34.60	2.21	7.71	6
A8	Atlantis	Quepos plateau	26/10/18	1,873	3.50	34.61	3.11	8.06	1, 3
F1	Falkor	The thumb**	10/1/19	1,072	4.54	34.58	1.22	7.69	4, 7
F2	Falkor	Parrita scar	11/1/19	1,419	3.35	34.61	2.08	7.67	4, 5
F3	Falkor	Rio bongo	13/1/19	659	14.41	34.93	1.50	7.60	4, 7
F4	Falkor	Subduction seep	14/1/19	3,474	1.88	34.66	4.20	7.71	4, 5
F5	Falkor	Seamount 5.5	15/1/19	1,540	3.00	34.62	2.64	7.70	4, 5
F6	Falkor	Seamount 7	16/1/19	1,320	4.11	34.59	1.80	7.67	4, 5
F7	Falkor	Coco Canyon	18/1/19	950	5.02	34.57	1.40	8.12	4, 5
F8	Falkor	Mound Jaguar**	25/1/19	1,903	2.43	34.63	3.13	7.75	4, 5

*1, AUV Sentry sensors; 2, HOV Alvin sensors; 3, HOV Alvin niskin bottle; 4, ROV SuBastian sensors; 5, ROV SuBastian niskin bottle; 6, RV Atlantis CTD; 7, RV Falkor CTD. **Seep areas.

We used the top 15 cm of the cores. Nearly one gram of the upper (1–2 cm), middle (6–7 cm), and lower (13–14 cm) parts of each core was deposited into a 1.5 ml tube, stored at −20°C on board the vessel and at −80°C in the laboratory. The sediment DNA was extracted with a DNA isolation kit (PowerSoil^®^, Qiagen, Carlsbad, CA, United States) following the manufacturer’s instructions. From some subsamples, unfortunately, it was not possible to obtain enough DNA for subsequent analyzes, so in total, we retrieved 40 DNA samples (out of the 48 possible) from the 16 cores sampled in both transects. The V7 and V8 regions of the 18S rRNA gene were amplified with primers FF390/FR1 (Vainio and Hantula, 2000), using the HotStarTaq Plus Master Mix Kit (Qiagen, Carlsbad, CA, United States). The PCR conditions consisted of 95°C for 3 min initial denaturation followed by 35 cycles at 95°C for 45 s, 53°C for 1 min, 72°C for 1 min, and a final extension at 72°C for 5 min. Multiple samples are pooled together in equal proportions based on their molecular weight and DNA concentrations. Pooled samples were purified using calibrated Ampure XP beads. The pooled and purified PCR product of nearly 350 bp were used to prepare illumina DNA library. Sequencing was performed at MR DNA^1^ (Shallowater, TX, United States) on a MiSeq sequencer with v3 2 × 250 nt chemistry (Illumina, San Diego, CA, United States).

We used the DADA2 pipeline version 1.16 to process the Illumina-sequenced paired-end fastq files and to generate a table of ASVs, which are higher-resolution analogs of the traditional OTUs (Callahan et al., 2016). Briefly, we removed primers and adapters, inspected the quality profiles of the reads, filtered and trimmed sequences with a quality score <30, estimated error rates, modeled and corrected amplicon errors and inferred the sequence variants. Then, we merged the forward and reverse reads to obtain the full denoised sequences, removed chimeras, and constructed the ASV table. To assign taxonomy to the ASVs we used the function *assignTaxonomy*, which is an implementation of a naive Bayesian classifier method using as input the set of sequences to be classified and a training set of reference sequences with known taxonomy, which in this case was Silva SSURef NR 132^2^ (Quast et al., 2013). The assignments were verified and further curated using the BLAST tool of NCBI Genbank. All ASVs that appeared only once in the dataset were discarded. The sequence data were deposited into the NCBI Sequence Read Archive under BioProject PRJNA632873 and BioSample accessions: SAMN14924417-SAMN14924456^3^.

Statistical analyses and their visualization were performed with the R statistical program (R-Core-Team, 2019) and the RStudio interface. Package Vegan v2.5-6 (Oksanen et al., 2020) was used to calculate alpha diversity estimators and, non-metric multidimensional scaling analyses (NMDS). Data tables with the ASV abundances were normalized into relative abundances and then converted into a Bray–Curtis similarity matrix. To determine if there were significant differences between the fungal community composition according to factors such as depth or transect, we used the non-parametric multivariate analysis of variance (PERMANOVA) and pairwise PERMANOVA (adonis2 function with 999 permutations). For the network analysis, we selected the 10 most abundant fungal ASVs, which corresponded to 82% of the total number of fungal sequences. We considered a valid co-occurrence event if the Spearman’s correlation coefficient was >0.5 (Junker, 2008). The resulting correlation matrix was converted into an undirected matrix. We used the R package igraph v1.2.4.2 to generate the network based on the Kamada–Kawai layout algorithm (Csardi and Nepusz, 2006).

The environmental data was collected from measurements performed in the water column overlying the sediment cores and for which various instruments and sensors were used (Table 1). Temperature and salinity data were obtained from the conductivity-temperature-depth (CTD) sensors on the HOV *Alvin* (CTD SeaBird SBE49) and ROV *SuBastian* (CTD Seabird FastCAT SBE49), which were also equipped with Niskin bottles for water sampling. There was a DO optode on the ROV *SuBastian* (Aanderaa 3841 O2 Optode) as well as the autonomous underwater vehicle (AUV) *Sentry* which was deployed over some of the sites during the 2018 *Atlantis* expedition. Niskin rosettes with attached CTDs were also deployed from the *Atlantis* and *Falkor* over the sites, and the *Falkor* CTD had a DO optode as well. DO data were compiled from a combination of these sources. DO data for the samples from the 2018 *Alvin* dives were derived from either the *Sentry* data (if available from the site) or calculated from a curve fitted from the closest CTD cast, typically, from the same site. DO data for the 2019 SuBastian push core samples was determined from SuBastian optode. The pH data were exclusively from the water samples obtained by the rosette deployed from the ship or the niskin bottles on the submersibles. Water samples were brought to room temperature and the pHT (total scale) was measured using an Orion 5 Star pH meter and glass electrode (ROSS Ultra pH/ATC Triode 8107BNUMD, Hamilton, NJ, United States) in triplicate within 4 h of collection (Dickson et al., 2007).

## Results and Discussion

**FIGURE 2:**
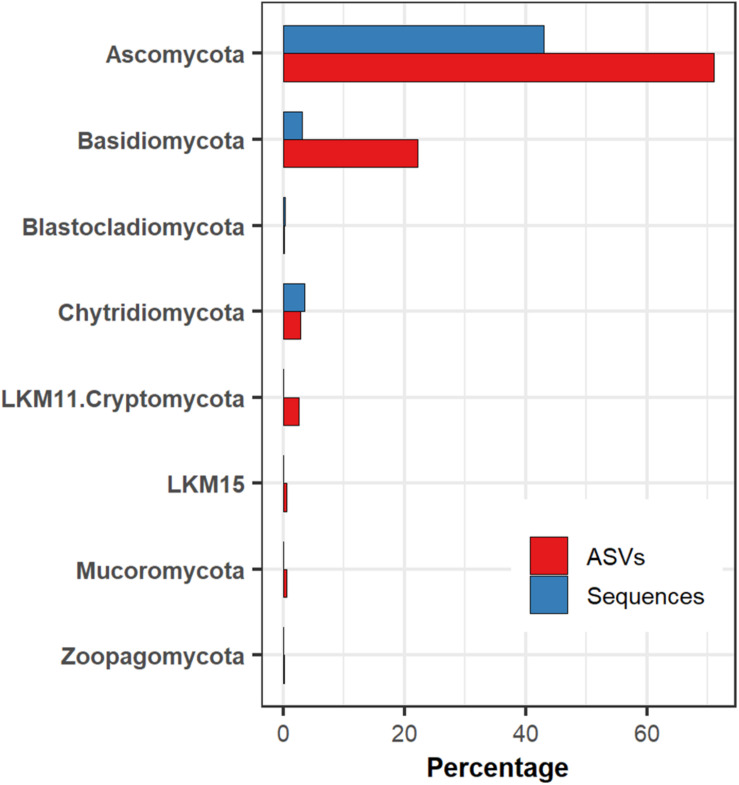
The relative abundance of fungal groups in deep-sea sediments of the Eastern Tropical Pacific of Costa Rica concerning the number of sequences and amplicon sequence variants (ASVs).

We determined the presence of 787 fungal ASV in marine sediments of the Eastern Tropical Pacific of Costa Rica, obtained from 16 locations (40 subsamples) along a bathymetric gradient from 380 to 3,474 m. Fungi represented 59.72% of the 2,746,436 sequences obtained from the specific primers used for the amplification of the V7-V8 region of the 18S rRNA gene. Ascomycota was the most abundant phylum, which represented 43% of all fungal sequences and 71% of the ASVs. The second most abundant fungal group was Basidiomycota, representing nearly 3% of the sequences but 22% of the ASVs. Most of the ASVs within Basidiomycota were assigned to the order Agaricales. Chytridiomycota represented the third most abundant fungal group, with 3.5% of the sequences and 2.79% of the ASVs. Other less frequent fungal groups observed in this ecosystem were, Blastocladiomycota, LKM11, LKM15, Mucoromycota, and Zoopagomycota (Figure 2).

**FIGURE 3:**
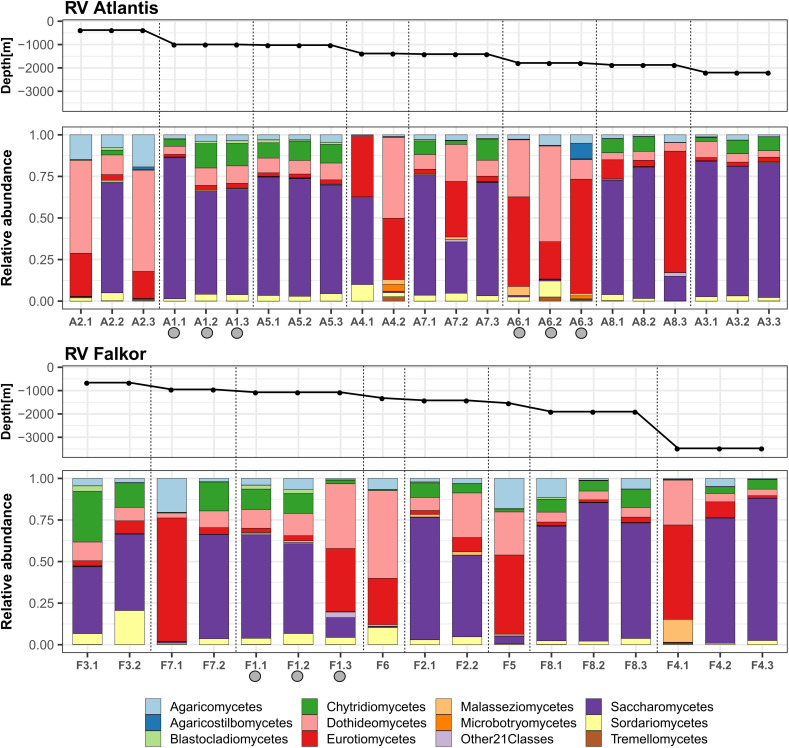
The relative abundance of fungi, at the taxonomic level of class, in deep-sea sediments of the eastern tropical Pacific of Costa Rica. The proportions within sampling points of the core subsamples for each of the cruise transects are shown. The samples were ordered according to the depth gradient. Gray circles indicate active methane seeps.

When analyzing the relative abundances at the class level, we detected a total of 32 classes in the deep-sea sediments, where Saccharomycetes was the most prominent in the majority of the samples. In samples where Saccharomycetes was dominant, they were typically accompanied by the presence of Chytridiomycetes. There was a second group of samples with high abundances of Eurotiomycetes, Dothideomycetes, and Agaricomycetes, but where Saccharomycetes were practically absent (Figure 3). This observation was consistent with positive correlations within each group. For example, the correlation calculated with the Spearman method between Saccharomycetes and Chytridiomycetes was 0.80, which implies that they were present in almost all the same samples and that they presented high values of their relative abundances. On the other hand, it was also determined that the correlations between the group dominated by Saccharomyces and the other dominated by filamentous fungi were negative (Supplementary Figure 1).

These results are consistent with those obtained, at the phylum level, in deep-sea sediments from places such as the Western and Central Pacific, the Mediterranean Sea or the São Paulo Plateau, which show Ascomycota as the most abundant group, together with the presence of Basidiomycota and Chytridiomycota in lower proportions (Li et al., 2016, 2019; Xu et al., 2016, 2019; Zhang et al., 2016; Nagano et al., 2017; Barone et al., 2018; Wang et al., 2019). However, this is the first work that shows, to our knowledge, the fungal class Saccharomycetes as the most abundant, and also highly correlated with Chytridiomycetes, in deep-sea sediments.

We also observed high variability in the fungal composition within the horizons of some samples. In this sense, the homogeneity or heterogeneity of the horizons could be related to the specific conditions of the sampled site, which include the geochemical characteristics of the region, the sedimentation time, as well as the microbiological activity. A limitation of this study is the lack of geochemical data on sediment cores, since the data on the environmental variables of the overlying water column are not sufficient to explain what is happening in the vertical gradient of sediments. Some studies have shown large variations in physicochemical conditions in the profile of deep-sea sediments (Roy et al., 2012; D’Hondt et al., 2015; Román et al., 2019). Therefore, it will be necessary to continue exploring in more detail the variations in fungal communities that occur along the vertical gradient of sediment profiles.

The samples of the deep-sea environment studied, characterized by high hydrostatic pressure, low temperatures, and the absence of light, presented an average richness of 75 fungal ASVs per sample (range 13–147), while the average value of the Shannon index was 1.77 (range 0.84–2.68). As with the community analyses, there were no significant differences in the alpha diversity estimations between depths and expeditions (Kruskal–Wallis, *p* > 0.05). The average value of the Pielou’s evenness was 0.42 (range 0.21–0.71), indicating a certain uniformity in the abundances of most of the observed ASVs (Supplementary Figure 2).

The genus *Metschnikowia* was the most abundant within the class Saccharomycetes and also the most abundant in the majority of the sediments analyzed. The genus *Metschnikowia* comprises single-celled budding yeasts known for its participation in fermentation processes and wine production, reported mainly in terrestrial environments (Kang et al., 2017; Wang et al., 2017; Pawlikowska et al., 2019). There are few references to the presence of this genus in deep waters, although its presence had been previously reported in subtropical Chinese seas, including the southern and northern Yellow Sea and the Bohai Sea (Li et al., 2016), but with lower abundances than those reported in this study. Also, we showed that this fungal genus was present in a wide depth gradient, from 380 to 3,474 m, indicating that it can be highly tolerant to gradients in temperature, DO, food supply, and the hydrostatic pressure associated with this change in depth. However, in six of the studied sediment cores *Metschnikowia* was almost absent, pointing more to microhabitat variability.

The most abundant genus within Chytridiomycetes was *Rhizophidium* which can function as parasite and decomposer (Letcher et al., 2006; Kagami et al., 2007; Frenken et al., 2017), while the most abundant genera of Eurotiomycetes and Dothideomycetes were *Aspergillus* and *Cladosporium*, respectively. Previous studies have shown that *Aspergillus* and *Penicillium* are common inhabitants of deep-sea sediments; likewise, the presence of yeasts in this ecosystem has been frequently detected, but mainly related to genera such as *Pichia*, *Cryptococcus*, *Malassezia*, and *Rhodotorula* (Takishita et al., 2006; Zhang et al., 2015; Nagano et al., 2016, 2017; Grossart et al., 2019). Within Agaricomycetes, the most abundant ASV had a percentage of identity of 98.73% with Armillaria, a saprophytic genus of wood that was particularly abundant in Coco Canyon (F7) at a depth of 950 m, which is also the site furthest from the coast. With the available information it is difficult to determine if this fungus, which is known to occur in terrestrial ecosystems, is active in these sediments.

Statistical analyzes, at the ASV level, did not show significant differences (PERMANOVA, *p* > 0.05) in the structure of fungal communities by depth, expeditions or between filtration/non-filtration areas. Neither according to variables of the overlying water columns of sediments such as pH, salinity and DO (Supplementary Table 1 and Supplementary Figure 3). For example, we showed that depth (and, consequently, hydrostatic pressure) does not have an apparent effect on the composition of communities, given the wide distribution range of species. In addition, the temperature, salinity, DO and pH values of the water column overlying the habitats of the two fungal clusters identified were similar (Table 2). Therefore, it seems that the conditions of the deep waters are not limiting for the growth of the fungi and that other factors, likely more related to the geochemistry of the sediments, can be influencing the composition of the communities.

**TABLE 2:** Depth, temperature, salinity, dissolved oxygen, and pH values of the water column overlying the habitats of the fungal clusters identified.

**Variable**	**Cluster 1 (yeast dominated)**	**Cluster 2 (filamentous forms)**
Depth (m)	380–1,788	659–3,474
Temperature (°C)	1.88–14.41	2.54–11.75
Salinity (PSU)	34.57–34.93	34.59–34.76
Dissolved oxygen (mg/L)	1.10–4.20	0.2–2.64
pH	7.60–8.06	7.61–7.70

As an empirical observation note, samples that contained a higher proportion of mud were the ones that exhibited a higher abundance of Saccharomycetes. In contrast, sandy samples showed higher abundances of Eurotiomycetes and Dothideomycetes, which are filamentous fungi. This observation suggests a possible relationship between fungal morphology and its ability to colonize substrates of different textures. For example, yeasts may directly depend on the type and concentrations of organic matter found in the habitat, but could also perform fermentation processes in muddy sediments (Takishita et al., 2006; Kutty and Philip, 2008; Zhang et al., 2015; Taube et al., 2018).

**FIGURE 4:**
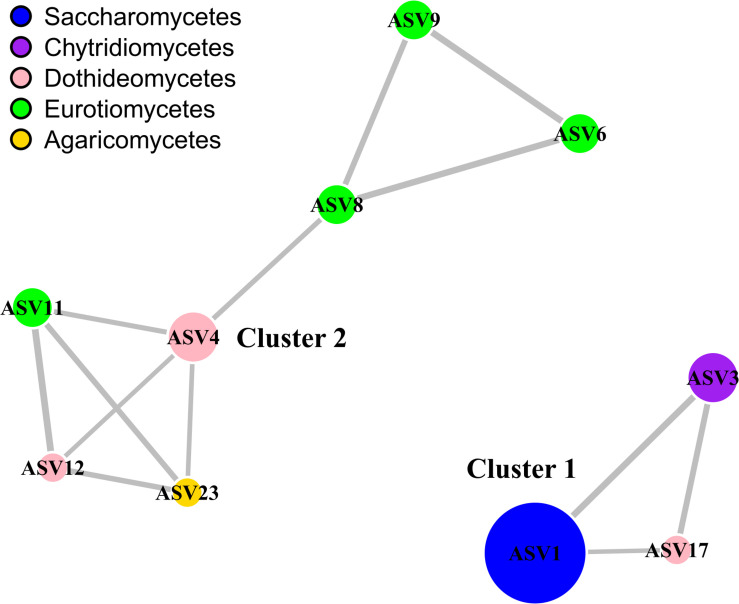
Network analysis highlighting the relationships between the most abundant fungal classes. The analysis is based on the 10 most abundant eukaryotic ASVs, which corresponded to 82% of the total number of fungal sequences. Only positive connections are shown. Colors of the nodes represent the taxonomic affiliation of the ASVs, while the size is proportional to their relative abundance. The width of the edges is proportional to the correlation value. The network was generated and visualized with package igraph. The taxonomic classification of the ASVs at the genus level is shown as follows: ASV1, *Metschnikowia*; ASV3, *Rhizophydium*; ASV4, *Cladosporium*; ASV6, *Aspergillus*; ASV8, *Aspergillus*; ASV9, *Aspergillus*; ASV11, *Exophiala*; ASV12, *Neophaeosphaeria*; ASV17, *Pseudocamarosporium*; ASV23, *Armillaria*.

We used network analysis to further explore possible relationships between the fungal groups that coexist in deep marine sediments of Costa Rica (Figure 4). This technique allowed us to visualize positive associations between the most abundant ASVs (representing 82% of the total sequences). We report a single co-occurrence and positive correlation between *Metschnikowia* and *Rhizophydium*. The association between these two taxa occurred regardless of the depth, location or conditions of the overlying water column. We have not found previous reports of the strong association between these two genera. We also report another group of co-occurring taxa that includes *Cladosporium* (Dothideomycetes), *Aspergillus* and *Exophiala* (Eurotiomycetes), and *Armillaria* (Agaricomycetes). The co-occurrence and high abundance of *Cladosporium* and *Aspergillus* is relatively common in deep-sea sediments (Li et al., 2019; Wang et al., 2019; Xu et al., 2019). However, with the available information it is difficult to determine whether the positive co-occurrence can be coincidental or can indicate a true positive interaction. Based on the results of the network analysis, as a hypothesis generating tool, we hypothesized that the fungi in both clusters can be carrying out mainly heterotrophic activities, but probably in sediments with different physicochemical conditions. The nature of the interactions within clusters should be further explored. Finally, we highlight the high prevalence of fungi in deep-sea sediments of the ETP of Costa Rica. To our knowledge, this is the first work showing a high abundance of *Metschnikowia* in deep-sea ecosystems. The high abundance of this type of yeasts should be further studied using cultivation-dependent methods to provide better insights into the physiology, genomic makeup, and their contributions to global biogeochemical processes. Since it was difficult to distinguish the association of specific environmental variables with variations in the composition of fungal communities, particularly in the two clusters identified, further research will be necessary to determine how fungal communities in deep-sea waters are structured as well as to determine their ecological role in the largest biome on the planet.

## Data Availability Statement

The datasets presented in this study can be found in online repositories. The sequence data were deposited into the NCBI Sequence Read Archive under BioProject PRJNA632873 and BioSample accessions: SAMN14924417–cSAMN14924456 (https://www.ncbi.nlm.nih.gov/bioproject/PRJNA632873).

## Author Contributions

KR-J, H-PG, EC, and JC designed the study and performed the analysis. JC and EC collected the samples. KR-J wrote the manuscript. All authors helped to revise the manuscript.

## Conflict of Interest

The authors declare that the research was conducted in the absence of any commercial or financial relationships that could be construed as a potential conflict of interest.

## Footnotes

1 www.mrdnalab.com

2 https://www.arb-silva.de/documentation/release-132/

3 https://www.ncbi.nlm.nih.gov/bioproject/PRJNA632873

## Supplementary Material

Supplementary Figure 1 Correlogram highlighting the most correlated classes of fungi in points in deep-sea sediments of the Eastern Tropical Pacific, based on Spearman correlation.

Supplementary Figure 2 Boxplots of the alpha diversity estimations of the sampling points in deep-sea sediments of the Eastern Tropical Pacific.

Supplementary Figure 3 Non-metric multidimensional scaling (NMDS) analyses of the fungal communities in deep-sea sediments. The analysis include 40 samples from sediments obtained in a bathymetric gradient (from a depth of 380 to 3,474 m) along two transects of about 1,500 km length in the Eastern Tropical Pacific of Costa Rica. The upper panel shows the analysis by transect and the lower the analysis by depth. The stress values of the NMDS and the *p* value of the PERMANOVA analyses are also shown.

Supplementary Table 1 Statistical analysis of the fungal community composition related to different variables. The PERMANOVA tests were performed using function adonis2 and implemented in Vegan package. Data were normalized by converting the ASV counts into relative abundances. Binning of continuous variables Depth, Temperature, Dissolved Oxygen and pH was performed with package Hmisc.

## References

[B1] Barone G. Rastelli E. Corinaldesi C. Tangherlini M. Danovaro R. Dell’Anno A. (2018). Benthic deep-sea fungi in submarine canyons of the Mediterranean Sea. *Prog. Oceanogr.* 168 57–64. 10.1016/j.pocean.2018.09.011

[B2] Batista-García R. A. Sutton T. Jackson S. A. Tovar-Herrera O. E. Balcázar-López E. del Rayo Sánchez-Carbente M. (2017). Characterization of lignocellulolytic activities from fungi isolated from the deep-sea sponge stelletta normani. *PLoS One* 12:e0173750. 10.1371/journal.pone.0173750 28339473 PMC5365110

[B3] Bochdansky A. B. Clouse M. A. Herndl G. J. (2017). Eukaryotic microbes, principally fungi and labyrinthulomycetes, dominate biomass on bathypelagic marine snow. *ISME J.* 11 362–373. 10.1038/ismej.2016.113 27648811 PMC5270556

[B4] Callahan B. J. McMurdie P. J. Rosen M. J. Han A. W. Johnson A. J. A. Holmes S. P. (2016). DADA2: high-resolution sample inference from Illumina amplicon data. *Nat. Methods* 13 581–583. 10.1038/nmeth.3869 27214047 PMC4927377

[B5] Cortés J. (2008). Historia de la investigación marina de la Isla del Coco, Costa Rica. *Rev. Biol. Trop.* 56 1–18.18624224

[B6] Cortés J. (2012). Marine biodiversity of an eastern tropical pacific oceanic island, Isla del coco, costa rica. *Rev. Biol. Trop.* 60 131–185.

[B7] Cortés J. (2016). “The pacific coastal and marine ecosystems,” in *Costa Rican Ecosystems*, ed. Kappelle M. (Chicago: The University of Chicago Press), 97–138. 10.7208/chicago/9780226121642.003.0005

[B8] Cortés J. (2019). “Isla del coco, costa rica, eastern tropical pacific,” in *Mesophotic Coral Ecosystems*, Vol 12, eds Y. Loya, K. Puglise, T. Bridge (Berlin: Springer), 465–475. 10.1007/978-3-319-92735-0_26

[B9] Csardi G. Nepusz T. (2006). The igraph software package for complex network research. *InterJournal Complex Syst.* 1695 1–9.

[B10] Dekas A. E. Connon S. A. Chadwick G. L. Trembath-Reichert E. Orphan V. J. (2016). Activity and interactions of methane seep microorganisms assessed by parallel transcription and FISH-NanoSIMS analyses. *ISME J.* 10 678–692. 10.1038/ismej.2015.145 26394007 PMC4817681

[B11] D’Hondt S. (2002). Metabolic activity of subsurface life in deep-sea sediments. *Science* 295 2067–2070. 10.1126/science.1064878 11896277

[B12] D’Hondt S. Inagaki F. Zarikian C. A. Abrams L. J. Dubois N. Engelhardt T. (2015). Presence of oxygen and aerobic communities from sea floor to basement in deep-sea sediments. *Nat. Geosci.* 8 299–304. 10.1038/ngeo2387

[B13] Dickson A. G. Sabine C. L. Christian J. R. (2007). *Guide to Best Practices for Ocean CO2 Measurements.* PICES Special Publication 3, 191. Available online at: https://www.nodc.noaa.gov/ocads/oceans/Handbook_2007.html

[B14] Drake H. Ivarsson M. (2018). The role of anaerobic fungi in fundamental biogeochemical cycles in the deep biosphere. *Fungal Biol. Rev.* 32 20–25. 10.1016/j.fbr.2017.10.001

[B15] Edgcomb V. P. Beaudoin D. Gast R. Biddle J. F. Teske A. (2011). Marine subsurface eukaryotes: the fungal majority. *Environ. Microbiol.* 13 172–183. 10.1111/j.1462-2920.2010.02318.x 21199255

[B16] Frenken T. Alacid E. Berger S. A. Bourne E. C. Gerphagnon M. Grossart H. P. (2017). Integrating chytrid fungal parasites into plankton ecology: research gaps and needs. *Environ. Microbiol.* 19 3802–3822. 10.1111/1462-2920.13827 28618196

[B17] Grossart H.-P. Van den Wyngaert S. Kagami M. Wurzbacher C. Cunliffe M. Rojas-Jimenez K. (2019). Fungi in aquatic ecosystems. *Nat. Rev. Microbiol.* 17 339–354. 10.1038/s41579-019-0175-8 30872817

[B18] Grossart H.-P. H.-P. P. Rojas-Jimenez K. (2016). Aquatic fungi: targeting the forgotten in microbial ecology. *Curr. Opin. Microbiol.* 31 140–145. 10.1016/j.mib.2016.03.016 27078576

[B19] Hassett B. T. Vonnahme T. R. Peng X. Jones E. B. G. Heuzé C. (2020). Global diversity and geography of planktonic marine fungi. *Bot. Mar.* 63 121–139. 10.1515/bot-2018-0113

[B20] Ivarsson M. Bengtson S. Neubeck A. (2016a). The igneous oceanic crust – Earth’s largest fungal habitat? *Fungal Ecol.* 20 249–255. 10.1016/j.funeco.2016.01.009

[B21] Ivarsson M. Schnürer A. Bengtson S. Neubeck A. (2016b). Anaerobic fungi: a potential source of biological H2 in the oceanic crust. *Front. Microbiol.* 7:674. 10.3389/fmicb.2016.00674 27433154 PMC4922220

[B22] Junker B. H. (2007). “Networks in Biology,” in *Analysis of Biological Networks*, eds Pan Y. Zomaya A. Y. Junker B. H. Schreiber F. 10.1002/9780470253489.ch1

[B23] Kagami M. de Bruin A. Ibelings B. W. Van Donk E. (2007). Parasitic chytrids: their effects on phytoplankton communities and food-web dynamics. *Hydrobiologia* 578 113–129. 10.1007/s10750-006-0438-z

[B24] Kang Y. M. Choi J. E. Komakech R. Park J. H. Kim D. W. Cho K. M. (2017). Characterization of a novel yeast species metschnikowia persimmonesis KCTC 12991BP (KIOM G15050 type strain) isolated from a medicinal plant, Korean persimmon calyx (diospyros kaki thumb). *AMB Express* 7:199. 10.1186/s13568-017-0503-1 29127501 PMC5681456

[B25] Kutty S. N. Philip R. (2008). Marine yeasts—a review. *Yeast* 25 465–483. 10.1002/yea.1599 18615863

[B26] Le Calvez T. Burgaud G. Mahé S. Barbier G. Vandenkoornhuyse P. Mahe S. (2009). Fungal diversity in deep-sea hydrothermal ecosystems. *Appl. Environ. Microbiol.* 75 6415–6421. 10.1128/AEM.00653-09 19633124 PMC2765129

[B27] Lenhart K. Bunge M. Ratering S. Neu T. R. Schüttmann I. Greule M. (2012). Evidence for methane production by saprotrophic fungi. *Nat. Commun.* 3:1046. 10.1038/ncomms2049 22948828

[B28] Letcher P. M. Powell M. J. Churchill P. F. Chambers J. G. (2006). Ultrastructural and molecular phylogenetic delineation of a new order, the *Rhizophydiales* (*Chytridiomycota*). *Mycol. Res.* 110 898–915. 10.1016/j.mycres.2006.06.011 16919432

[B29] Levin L. A. Mendoza G. F. Grupe B. M. Gonzalez J. P. Jellison B. Rouse G. (2015). Correction: biodiversity on the rocks: macrofauna inhabiting authigenic carbonate at costa rica methane seeps. *PLoS One* 10:e0136129. 10.1371/journal.pone.0136129 26274609 PMC4537242

[B30] Levin L. A. Orphan V. J. Rouse G. W. Rathburn A. E. Ussler W. Cook G. S. (2012). A hydrothermal seep on the Costa Rica margin: middle ground in a continuum of reducing ecosystems. *Proc. R. Soc. B Biol. Sci.* 279 2580–2588. 10.1098/rspb.2012.0205 22398162 PMC3350710

[B31] Li W. Wang M. Burgaud G. Yu H. Cai L. (2019). Fungal community composition and potential depth-related driving factors impacting distribution pattern and trophic modes from epi- to abyssopelagic zones of the Western Pacific Ocean. *Microb. Ecol.* 78 820–831. 10.1007/s00248-019-01374-y 30993370

[B32] Li W. Wang M. M. Wang X. G. Cheng X. L. Guo J. J. Bian X. M. (2016). Fungal communities in sediments of subtropical Chinese seas as estimated by DNA metabarcoding. *Sci. Rep.* 6 26528. 10.1038/srep26528 27198490 PMC4873734

[B33] Lizano O. (2001). Batimetria de la plataforma insular alrededor de las Isla del coco, costa rica. *Rev. Biol. Trop.* 49(Suppl), 163–170. 15264530

[B34] López-García P. Philippe H. Gail F. Moreira D. (2003). Autochthonous eukaryotic diversity in hydrothermal sediment and experimental microcolonizers at the mid-atlantic ridge. *Proc. Natl. Acad. Sci. U.S.A.* 100 697–702. 10.1073/pnas.0235779100 12522264 PMC141059

[B35] Manohar C. S. Raghukumar C. (2013). Fungal diversity from various marine habitats deduced through culture-independent studies. *FEMS Microbiol. Lett.* 341 69–78. 10.1111/1574-6968.12087 23363246

[B36] Morales S. E. Biswas A. Herndl G. J. Baltar F. (2019). Global Structuring of phylogenetic and functional diversity of pelagic fungi by depth and temperature. *Front. Mar. Sci.* 6:131 10.3389/fmars.2019.00131

[B37] Nagahama T. Takahashi E. Nagano Y. Abdel-Wahab M. A. Miyazaki M. (2011). Molecular evidence that deep-branching fungi are major fungal components in deep-sea methane cold-seep sediments. *Environ. Microbiol.* 13 2359–2370. 10.1111/j.1462-2920.2011.02507.x 21605311

[B38] Nagano Y. Konishi M. Nagahama T. Kubota T. Abe F. Hatada Y. (2016). Retrieval of deeply buried culturable fungi in marine subsurface sediments, Suruga-Bay, Japan. *Fungal. Ecol.* 20 256–259. 10.1016/j.funeco.2015.12.012

[B39] Nagano Y. Miura T. Nishi S. Lima A. O. Nakayama C. Pellizari V. H. (2017). Fungal diversity in deep-sea sediments associated with asphalt seeps at the São Paulo Plateau. *Deep Sea Res. Part II Top. Stud. Oceanogr.* 146 59–67. 10.1016/j.dsr2.2017.05.012

[B40] Nagano Y. Nagahama T. (2012). Fungal diversity in deep-sea extreme environments. *Fungal Ecol.* 5 463–471. 10.1016/j.funeco.2012.01.004 22222832

[B41] Nealson K. H. Inagaki F. Takai K. (2005). Hydrogen-driven subsurface lithoautotrophic microbial ecosystems (SLiMEs): do they exist and why should we care? *Trends Microbiol.* 13 405–410. 10.1016/j.tim.2005.07.010 16054814

[B42] Oksanen J. Blanchet F. G. Friendly M. Kindt R. Legendre P. McGlinn D. (2020). *vegan: Community Ecology Package (R package version 2.5–6).*

[B43] Pachiadaki M. G. Rédou V. Beaudoin D. J. Burgaud G. Edgcomb V. P. (2016). Fungal and prokaryotic activities in the marine subsurface biosphere at Peru margin and Canterbury Basin inferred from RNA-based analyses and microscopy. *Front. Microbiol.* 7:846. 10.3389/fmicb.2016.00846 27375571 PMC4899926

[B44] Pawlikowska E. James S. A. Breierova E. Antolak H. Kregiel D. (2019). Biocontrol capability of local *Metschnikowia* sp. isolates. *Antonie Van Leeuwenhoek* 112 1425–1445. 10.1007/s10482-019-01272-w 31111331 PMC6748895

[B45] Protti M. González V. Freymueller J. Doelger S. (2012). Isla del Coco, on Cocos Plate, converges with Isla de San Andrés, on the Caribbean Plate, at 78mm/yr. *Rev. Biol. Trop.* 60 33–41.

[B46] Quast C. Pruesse E. Yilmaz P. Gerken J. Schweer T. Yarza P. (2013). The SILVA ribosomal RNA gene database project: improved data processing and web-based tools. *Nucleic Acids Res.* 41 D590–D596. 10.1093/nar/gks1219 23193283 PMC3531112

[B47] R-Core-Team (2019). *R: A Language and Environment for Statistical Computing*.

[B48] Rojas W. Alvarado G. E. (2012). Marco geológico y tectónico de la Isla del Coco y la región marítima circunvecina, Costa Rica zone off the central Pacific coast of Costa Rica. *Rev. Biol. Trop.* 60 15–32.

[B49] Rojas-Jiménez K. (2018). Microorganismos del corredor marino isla del coco-galápagos: diversidad funcional y de especies. *Rev. Tecnol. en Marcha* 31 157–166. 10.18845/tm.v31i4.3974

[B50] Román S. Ortiz-Álvarez R. Romano C. Casamayor E. O. Martin D. (2019). Microbial community structure and functionality in the deep sea floor: evaluating the causes of spatial heterogeneity in a submarine canyon system (NW Mediterranean, Spain). *Front. Mar. Sci.* 6:108 10.3389/fmars.2019.00108

[B51] Roy H. Kallmeyer J. Adhikari R. R. Pockalny R. Jorgensen B. B. D’Hondt S. (2012). Aerobic microbial respiration in 86-million-year-old deep-sea red clay. *Science* 336 922–925. 10.1126/science.1219424 22605778

[B52] Rusch D. B. Halpern A. L. Sutton G. Heidelberg K. B. Williamson S. Yooseph S. (2007). The sorcerer II global ocean sampling expedition: northwest atlantic through Eastern Tropical Pacific. *PLoS Biol.* 5:e77. 10.1371/journal.pbio.0050077 17355176 PMC1821060

[B53] Sahling H. Masson D. G. Ranero C. R. Hühnerbach V. Weinrebe W. Klaucke I. (2008). Fluid seepage at the continental margin offshore Costa Rica and southern Nicaragua. *Geochem. Geophys. Geosyst.* 9 1–22. 10.1029/2008GC001978

[B54] Takishita K. Tsuchiya M. Reimer J. D. Maruyama T. (2006). Molecular evidence demonstrating the basidiomycetous fungus *Cryptococcus curvatus* is the dominant microbial eukaryote in sediment at the Kuroshima Knoll methane seep. *Extremophiles* 10 165–169. 10.1007/s00792-005-0495-7 16341819

[B55] Taube R. Ganzert L. Grossart H.-P. Gleixner G. Premke K. (2018). Organic matter quality structures benthic fatty acid patterns and the abundance of fungi and bacteria in temperate lakes. *Sci. Total Environ.* 61 469–481. 10.1016/j.scitotenv.2017.07.256 28818662

[B56] Vainio E. J. Hantula J. (2000). Direct analysis of wood-inhabiting fungi using denaturing gradient gel electrophoresis of amplified ribosomal DNA. *Mycol. Res.* 104 927–936. 10.1017/S0953756200002471

[B57] Wang C. Liu Y. Zhang T. Lu C. Liu Y. Zhang D. (2017). Metschnikowia persici sp. nov., a novel protease-producing yeast species from China. *Curr. Microbiol.* 74 365–370. 10.1007/s00284-017-1194-1 28168603

[B58] Wang Z.-P. Liu Z.-Z. Wang Y.-L. Bi W.-H. Liu L. Wang H.-Y. (2019). Fungal community analysis in seawater of the mariana trench as estimated by Illumina HiSeq. *RSC Adv.* 9 6956–6964. 10.1039/C8RA10142F PMC9061085 35518513

[B59] Xu W. Gao Y. Gong L. Li M. Pang K.-L. Luo Z.-H. (2019). Fungal diversity in the deep-sea hadal sediments of the yap trench by cultivation and high throughput sequencing methods based on ITS rRNA gene. *Deep Sea Res. Part I Oceanogr. Res. Pap.* 145 125–136. 10.1016/j.dsr.2019.02.001

[B60] Xu W. Guo S. Pang K.-L. Luo Z.-H. (2017). Fungi associated with chimney and sulfide samples from a South Mid-Atlantic Ridge hydrothermal site: distribution, diversity and abundance. *Deep Sea Res. Part I Oceanogr. Res. Pap.* 123 48–55. 10.1016/j.dsr.2017.03.004

[B61] Xu W. Luo Z.-H. Guo S. Pang K.-L. (2016). Fungal community analysis in the deep-sea sediments of the Pacific Ocean assessed by comparison of ITS, 18S and 28S ribosomal DNA regions. *Deep Sea Res. Part I Oceanogr. Res. Pap.* 109 51–60. 10.1016/j.dsr.2016.01.001

[B62] Xu Z. Wang M. Wu W. Li Y. Liu Q. Han Y. (2018). Vertical distribution of microbial eukaryotes from surface to the hadal zone of the mariana trench. *Front. Microbiol.* 9:2023. 10.3389/fmicb.2018.02023 30210485 PMC6120995

[B63] Zhang T. Fei Wang N. Qin Zhang Y. Yu Liu H. Yan Yu L. (2015). Diversity and distribution of fungal communities in the marine sediments of Kongsfjorden, Svalbard (High Arctic). *Sci. Rep.* 5:14524. 10.1038/srep14524 26494429 PMC4615975

[B64] Zhang X. Tang G. Xu X. Nong X. Qi S.-H. (2014). Insights into deep-sea sediment fungal communities from the east indian ocean using targeted environmental sequencing combined with traditional cultivation. *PLoS One* 9:e109118. 10.1371/journal.pone.0109118 25272044 PMC4182876

[B65] Zhang X.-Y. Wang G.-H. Xu X.-Y. Nong X.-H. Wang J. Amin M. (2016). Exploring fungal diversity in deep-sea sediments from Okinawa Trough using high-throughput Illumina sequencing. *Deep Sea Res. Part I Oceanogr. Res. Pap.* 116 99–105. 10.1016/j.dsr.2016.08.004

