# Supplementary material for: Fungal Communities in Sediments Along a Depth Gradient in the Eastern Tropical Pacific
Source: Front Microbiol. 2020 Nov 6;11:575207. doi: 10.3389/fmicb.2020.575207 (PMC7681244; doi:10.3389/fmicb.2020.575207)
Supplement: Supplementary Figure 1 — Correlogram highlighting the most correlated classes of fungi in points in deep-sea sediments of the Eastern Tropical Pacific, based on Spearman correlation. [file Data_Sheet_1.docx]

**Supplementary Table 1**.

| **Variable** | **Comparison** | **R2** | **F.Model** | **p.value** |
| --- | --- | --- | --- | --- |
| Expedition | Atlantlis vrs Falkor | 0.02092 | 0.8119 | 0.4555 |
| Feature | Seep vrs no-seep | 0.05148 | 1.0041 | 0.3506 |
| Salinity | All (16 locations) | 0.01851 | 0.7165 | 0.5275 |
| Depth | All (16 locations) | 0.02929 | 1.1467 | 0.2697 |
|  | 380-1419 m vrs 1419-3474m | 0.02233 | 0.8678 | 0.4046 |
| Temperature | All (16 locations) | 0.02843 | 1.1121 | 0.2947 |
|  | 1.88-3.5 vrs 3.50-14.4 | 0.02003 | 0.7765 | 0.4585 |
| Dissolved Oxygen | All (16 locations) | 0.03454 | 1.3597 | 0.2118 |
|  | 0.20-2.21 vrs 2.21-4.20 mg/L | 0.02428 | 0.9456 | 0.3686 |
| pH | All (16 locations) | 0.03003 | 1.1765 | 0.2607 |
|  | 7.60-7.71 vrs 7.71-8.12 | 0.02863 | 1.12 | 0.2697 |
