# Supplementary figures and images for: Fungal Communities in Sediments Along a Depth Gradient in the Eastern Tropical Pacific
Source: Front Microbiol. 2020 Nov 6;11:575207. doi: 10.3389/fmicb.2020.575207 (PMC7681244; doi:10.3389/fmicb.2020.575207)

**Supplementary Figure 1**


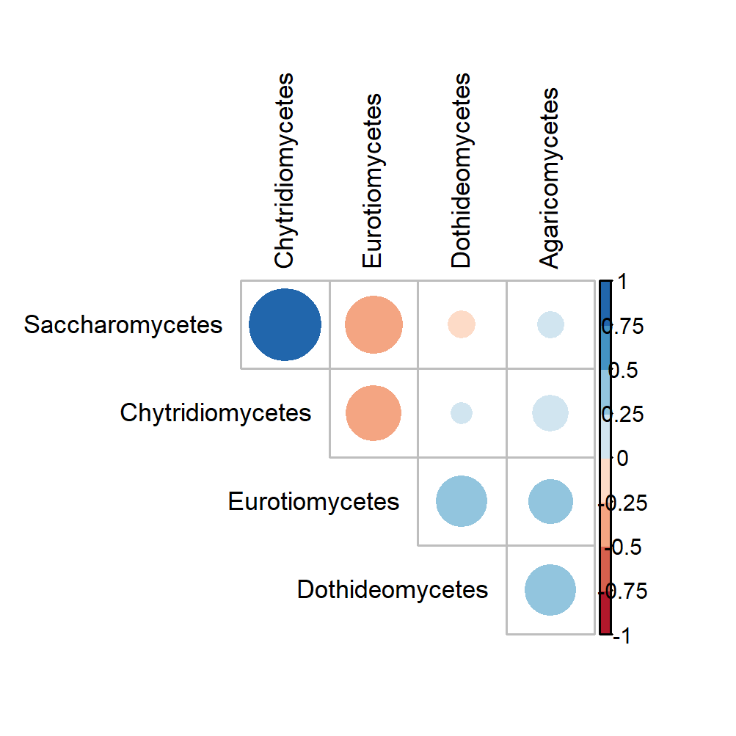


**Supplementary Figure 2**


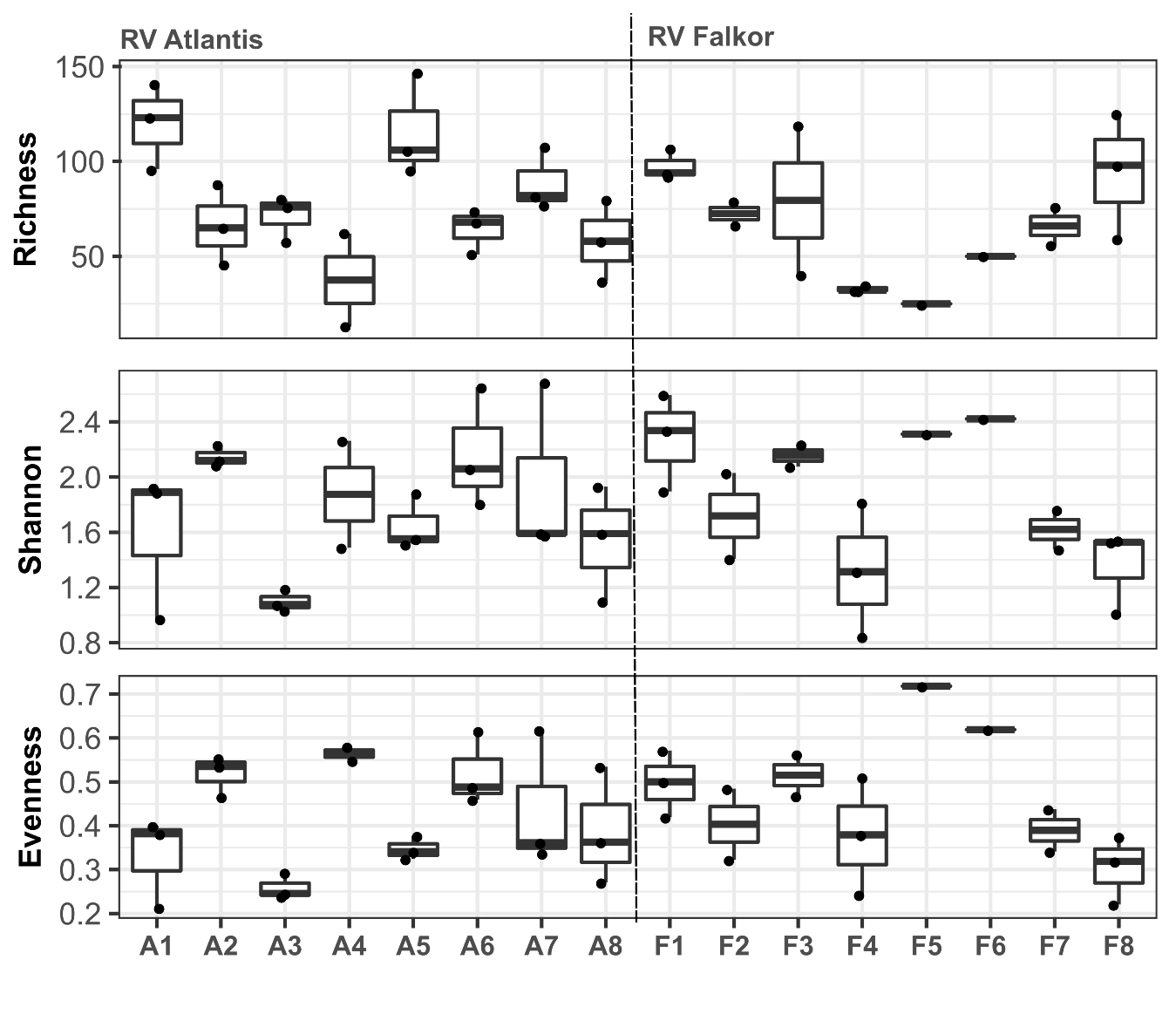


**Supplementary Figure 3**


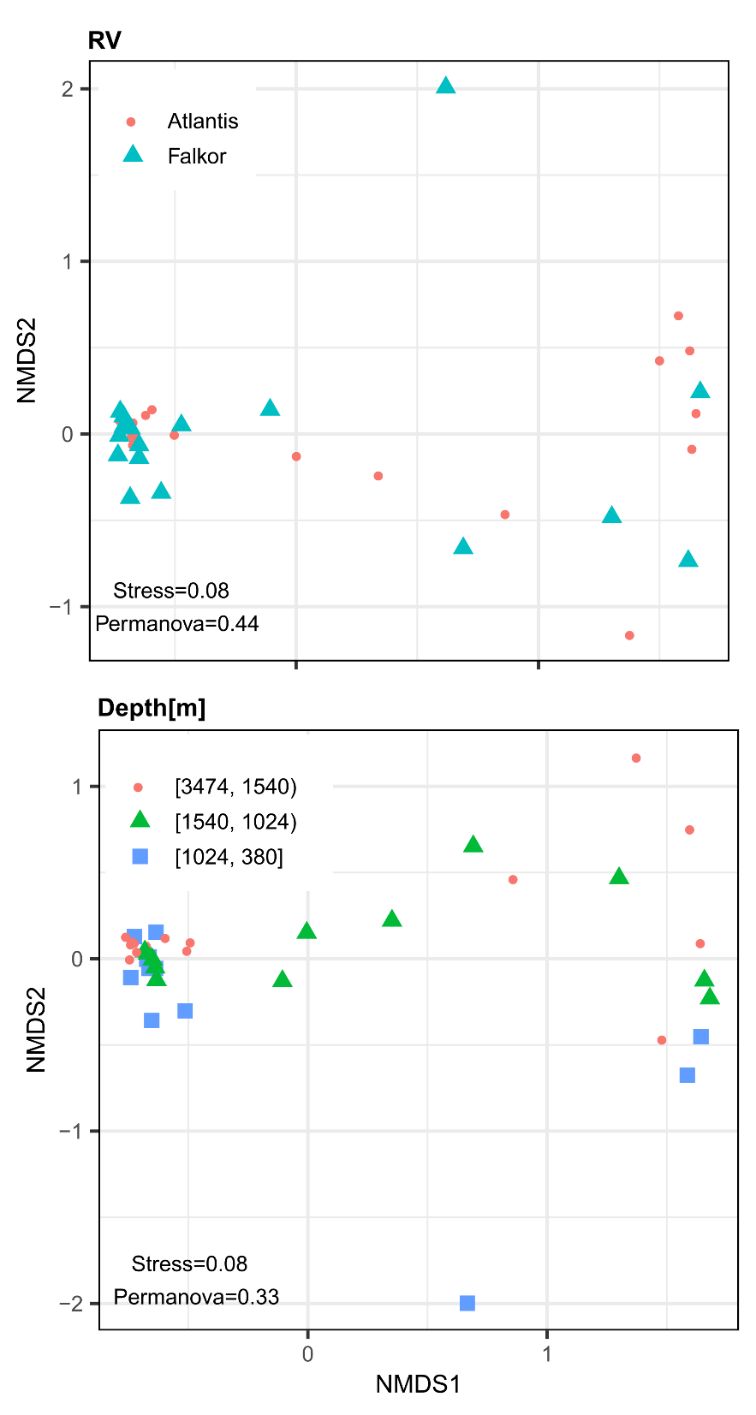

Supplement: Supplementary Table 1 — Statistical analysis of the fungal community composition related to different variables. The PERMANOVA tests were performed using function adonis2 and implemented in Vegan package. Data were normalized by converting the ASV counts into relative abundances. Binning of continuous variables Depth, Temperature, Dissolved Oxygen and pH was performed with package Hmisc. [file Data_Sheet_2.docx]
